# Synergistic use of ICESat-2 lidar data and Sentinel-2 imagery for assessing hurricane-driven forest changes

**DOI:** 10.1007/s10661-025-14749-1

**Published:** 2025-11-07

**Authors:** Ajay Gautam, Lana L. Narine, Christopher J. Anderson, Richard Cristan

**Affiliations:** College of Forestry, Wildlife and Environment, Auburn University, 3301 FWS Building, 602 Duncan Drive, Auburn, AL 36849 USA

**Keywords:** Random Forest, XGBoost, Canopy height, Canopy cover

## Abstract

Coastal forests worldwide are vulnerable to hurricanes, which cause significant canopy loss and long-term disruption of ecosystem services. Scalable methods for assessing hurricane-driven forest damage are critical for ecosystem recovery, yet conventional field-based approaches are time-consuming, costly and challenging to acquire over large areas. The Ice Cloud and land Elevation Satellite-2 land and vegetation height product (ATL08) provides three-dimensional information, and its capability of measuring forest structure is already well demonstrated. However, its integration with satellite imagery for mapping hurricane-induced canopy height changes remains limited. In this study, we analyzed coastal forest canopy losses and thematic transitions using ICESat-2, Sentinel-2, and ancillary predictors. We used Random Forest (RF) and Extreme Gradient Boosted (XGB) regression models to extrapolate ATL08 pre-hurricane canopy heights and applied the better-performing model for post-hurricane mapping. The resulting canopy height maps were combined with existing land cover products to assess structural and thematic transitions across the impacted landscape. RF outperformed XGB (R^2^ = 0.44, RMSE = 4.30 m vs. R^2^ = 0.41, RMSE = 4.76 m). Landcover shifts included transitions from evergreen to herbaceous, scrub, and barren classes, and from woody wetlands to emergent herbaceous wetlands, with mean canopy height losses of 2.3–5.2 m. Canopy cover analysis showed dense (> 60%) and sparse (< 30%) cover experienced greatest losses (up to 8.3 m), while moderate covers (30–60%) were resilient. This study demonstrates the potential of integrating ICESat-2 and Sentinel-2 for assessing structural and thematic changes and informing adaptive strategies in hurricane-prone coastal ecosystems.

## Introduction

Forests are complex ecological systems that function as vital carbon reservoirs and play a crucial role in global climate regulation, biodiversity conservation, and ecosystem stability (Barry et al., 1993; Gresham et al., 1991; Xi et al., 2008). In the United States (US), forests contribute significantly to the national economy, estimated at over $300 billion turnover annually from timber and manufacturing sectors, of which the southern states account for approximately 45% of the total value (Alvarez, 2020). Despite their ecological and economic significance, these forests are vulnerable to extreme weather events such as hurricanes, storms, wildfires, and droughts which can lead to extensive canopy loss, structural damage, and long-term shifts in forest composition (Cole et al., 2021; Seidl et al., 2011, 2017; Xi et al., 2018).

Post-hurricane assessments are critical for quantifying structural forest losses, particularly reductions in vertical forest structure caused by sustained impacts from breakage, mortality, disease, succession, and long-term stress (Barry et al., 1993; Seidl et al., 2017; Sharma et al., 2021) Assessing changes in forest structure, especially canopy height, is essential for tracking forest recovery, productivity, and long-term ecosystem stability (Blackman & Yuan, 2020; Favrichon et al., 2025). Determining canopy height-related changes is fundamental for understanding ecosystem changes and quantifying the severity of such damages (Bloom et al., 2023; Favrichon et al., 2025; Leitold et al., 2021). However, a major challenge in post-hurricane assessment is the limited availability of multi-temporal high-resolution forest structural information.

Traditional forest damage assessments have relied heavily on field surveys and permanent plot inventories, which are labor-intensive, time-consuming, and costly (Blackman & Yuan, 2020). For example, following Hurricane Michael in 2018, the U.S. Forest Service’s Forest Inventory and Analysis (FIA) program took two years to survey just 5367 trees, a fraction of the estimated 2.4 billion trees affected by the storm (Brandeis et al., 2022). Key challenges in understanding hurricane impacts on forests lie in their sustained after-effects on forest health and the lack of comprehensive assessments of canopy height changes across the spatial extent of hurricane damage (Cohen et al., 2024). Although structural information from FIA plots is valuable, these plots are typically revisited on a decadal basis and often reflect cumulative rather than event-specific changes (Klesse et al., 2018), making this information insufficient for capturing the impacts of a single extreme event. Further, accurate post-disturbance assessments at the regional level are critical for understanding the extent and severity of such impacts, informing forest recovery and management policies, and guiding long-term resilience strategies (Cole et al., 2021; Ochoa & Urbina-Cardona, 2017).

Remote sensing (RS) technologies have facilitated broader, and more frequent assessments of forest disturbance, utilizing radar, multispectral, hyperspectral and lidar sensors. Low, moderate, and high-resolution satellite imagery, including MODIS (500 m), Landsat (30 m), and Sentinel-2 (10 m–20 m), as well as very high-resolution airborne and Unmanned Aerial Vehicle (UAV)-based data, have been widely applied to hurricane damage assessments (Asif et al., 2018; Feng et al., 2018; Hu & Smith, 2018; Turner et al., 2023; Wang & Xu, 2010). Multi-temporal observations from Landsat-8 and Sentinel-2, and derived vegetation indices (VIs) have proven particularly effective in capturing post-hurricane vegetation dynamics (Aljahdali et al., 2021; Chambers et al., 2007; Feng et al., 2020; Negrón-Juárez et al., 2014; Wang & Xu, 2010). For instance, Hu and Smith (2018) analyzed vegetation changes following Hurricane Maria in Dominica and Puerto Rico using time-series imagery and vegetation indices derived from Landsat-8 and Sentinel-2. Authors used Spectral Mixture Analysis (SMA) to estimate proportions of green vegetation (gv) and non-photosynthetic vegetation (npv) based on reflectance properties (Chambers et al., 2007; Negrón-Juárez et al., 2014). Feng et al. (2020) also applied SMA using Landsat and MODIS data to assess post-Maria canopy damage in Puerto Rico, revealing a 34% transition from gv to npv. Such techniques have proven valuable for detecting forest disturbances and monitoring recovery across a range of spatial and temporal scales.

Despite their utility, optical RS approaches are inherently limited to two-dimensional (2D) representations, constraining their ability to capture vertical forest structure attributes, such as canopy height. This limits the accuracy of damage severity estimates, especially in cases of partial canopy loss or early regrowth stages. These limitations highlight a need for scalable, high-resolution methods capable of capturing three-dimensional (3D) forest structural changes across broader spatial extents, particularly following major disturbance events.

Light Detection and Ranging (lidar) provides essential 3D information on forest structure, enabling accurate measurements of forest canopy height and terrain elevation (Anderson et al., 2006; Dassot et al., 2011; Lefsky et al., 2002). Although extensive airborne lidar datasets exist in the US, they are often temporally sparse and subject to variability in sensor specifications, acquisition protocols, and spatial coverage (Besic et al., 2025). These limitations hinder their effectiveness for systematic, long-term monitoring of forest height. In contrast, data from current or ongoing spaceborne lidar missions may offer near-global coverage and repeat observations, making data from this platform particularly valuable for detecting and assessing large-area forest disturbances, including those caused by hurricanes.

This context highlights the importance of NASA’s ongoing spaceborne lidar mission, the Ice, Cloud, and land Elevation Satellite-2 (ICESat-2), which provides consistent, near global-scale along-track vegetation height measurements (Neumann et al., 2019). NASA’s first satellite-based laser altimetry mission for Earth, ICESat (2003–2009), employed the Geoscience Laser Altimeter System (GLAS), which enabled the global-scale estimation of canopy height (Wang et al., 2011). GLAS data were successfully applied to quantify vegetation loss from events like Hurricane Katrina, revealing strong correlations between canopy height reductions and storm wind intensity (Dolan et al., 2011). ICESat-2 was launched in 2018, with substantial improvements in spatial resolution and vertical accuracy over ICESat through a photon-counting laser instrument; Advanced Topographic Laser Altimeter System (ATLAS) (Abdalati et al., 2010; Markus et al., 2017).

ATLAS emits laser pulses that are split into six beams, three strong and three weak, to provide dense elevation sampling along the satellite’s orbital path (Markus et al., 2017; Neuenschwander & Pitts, 2019; Neuenschwander et al., 2022). ATLAS records individual photon returns from Earth’s surface, enabling fine-scale vertical profiling of Earth’s geophysical characteristics. Although three of the mission’s primary objectives are focused on monitoring the cryosphere, the fourth objective is focused on characterization of global vegetation structure (Abdalati et al., 2010; Markus et al., 2017). Repeated and crossover ICESat-2 tracks have shown potential for observing flood level changes (Thomas et al., 2023), land ice height changes (Smith et al., 2019), which provides evidence for analyzing multi-temporal ICESat-2 tracks to determine structural canopy changes resulting from disturbances like hurricanes. ATL08 is the mission’s dedicated vegetation product that provides key vegetation structural metrics such as canopy height and terrain elevation (Neuenschwander & Magruder, 2019; Neuenschwander & Pitts, 2019). ATL08 has shown strong agreement with reference airborne lidar data (Root Mean Square Errors (RMSE) < 3.2 m, correlation coefficient (R) > 0.82) for canopy height extraction, affirming ICESat-2’s potential for broad-scale forest structural analysis (Li et al., 2020; Yu et al., 2024).

Although ICESat-2 provides highly accurate vegetation structure data, a major limitation lies in its spatially discontinuous sampling pattern, as it collects data only along narrow orbital tracks. To overcome this limitation, studies have explored the integration of ICESat-2 ATL08 canopy metrics with spatially continuous datasets, such as multispectral satellite imagery (e.g., Sentinel-2, Landsat), vegetation indices (VIs), topographic variables, and land cover datasets, using statistical, geostatistical, machine learning (ML), or deep learning (DL) approaches. These integrated methods have proven effective in generating high-resolution (10 m–250 m) gridded forest structure maps across large areas, especially in regions affected by disturbance events (Coops et al., 2021; Favrichon et al., 2025; Queinnec et al., 2024).

ML models have become increasingly prominent in remote sensing applications due to their capacity to handle large and complex datasets (Li et al., 2020; Ma et al., 2019; Martinez-Amaya et al., 2022), with significant applications in forestry research (H. Liu et al., 2021a, 2021b; Ma et al., 2019; Thapa et al., 2023). Among these, Random Forest (RF) algorithm remains one of the most widely used models due to its robustness against overfitting and ability to manage multicollinearity among predictor variables (Cutler et al., 2007). Other widely used ML models like support vector machines (SVM), extreme gradient boosting (XGB), and k-nearest neighbors (KNN) and Deep learning architectures such as deep neural networks (DNNs) and convolutional neural networks (CNNs), have also demonstrated high accuracy in predicting canopy height, cover, and other structural attributes (Li et al., 2020; Luo et al., 2019; Narine et al., 2023; Pourshamsi et al., 2018).

Geostatistical and ML models have demonstrated considerable potential for upscaling ICESat-2-derived canopy height and related vegetation parameters (Malambo & Popescu, 2024; Malambo et al., 2022; Pan et al., 2022; Tiwari & Narine, 2022). For instance, Narine et al. (2023) used RF for integrating ICESat-2 parameters with 30-m gap-filled Landsat surface reflectance (Moreno-Martinez et al., 2020), and derived VIs to generate ICESat-2-based canopy cover maps over southeastern US research sites. The results showed moderate to high accuracy (R = 0.57–0.78 and R^2^ = 0.81) when compared with airborne lidar and National Land Cover Database (NLCD) reference data. In another study, Malambo et al. (2022) extrapolated ICESat-2 along-track height estimates using Landsat and Landscape Fire and Resource Management Planning Tools (LANDFIRE) predictors with a gradient boosting model to produce regional-scale canopy height maps in eastern Texas (R^2^ = 0.69, MAE = 2.09 m). At broader scales, Malambo and Popescu (2024) used tuned hyperparameters in an extreme gradient boosted (XGB) model to produce a conterminous US canopy height map, reporting good accuracy (R^2^ = 0.76 and mean bias = 0.1 m with test data). Lang et al. (2019) and Malambo et al. (2022) applied gradient boosted regression models to produce ICESat-2-derived canopy height maps.

While these advancements have led to the development of large-area spaceborne lidar-derived canopy height products (e.g., Malambo & Popescu, 2024; Potapov et al., 2021), several studies emphasize that such generalized models may not capture local-scale canopy height variations accurately (Favrichon et al., 2025). This limitation underscores the importance of producing disturbance-specific canopy height maps, especially in the context of hurricane impacts, where vegetation change patterns are highly heterogeneous. In such cases, local models have shown greater effectiveness in detecting structural change (Fogel et al., 2024), reinforcing the need for fine-scale modeling tailored to specific disturbance events and ecosystem context. The spatial heterogeneity of hurricane impacts, particularly treefall, partial canopy loss, and mortality-induced height changes, necessitates the use of locally trained machine learning models that can inform changes in canopy heights across the overall extent of damage. ICESat-2 data have recently been applied in a range of vegetation studies, including estimating forest biomass (e.g., Bueno et al., 2025; Jiang et al., 2022; Liu et al., 2024; Narine et al., 2019, 2020; Song et al., 2022; Tiwari et al., 2025) validating tree height measurements (Huang et al., 2023; Malambo & Popescu, 2024; Mansouri et al., 2024; Tiwari & Narine, 2022; Vatandaslar et al., 2022; Yu et al., 2024), and analyzing forest degradation and temporal changes in canopy structure due to disturbances such as wildfire and drought (Chen et al., 2023; Favrichon et al., 2025; Hosseiny et al., 2024; Konduri et al., 2023; Liu et al., 2023; Mehmood et al., 2025). Despite these advances, the use of multi-temporal ICESat-2 observations to detect canopy height changes resulting specifically from hurricane disturbances remains limited. This study addresses that gap by combining ICESat-2 canopy metrics with Sentinel-2 imagery and ancillary geospatial data to develop localized models to capture the extent and intensity of hurricane-induced forest structural changes.

The goal of this study is to establish a framework for assessing hurricane-induced structural changes in coastal forests by leveraging multi-temporal ICESat-2 data with other satellite-based datasets to assess hurricane-driven forest changes, considering both forest structure and land cover. Using Hurricane Sally (2020) as a case study, this study serves to understand the utility of ICESat-2 for detecting hurricane-driven, multi-temporal changes in forest canopy and evaluate the effectiveness ICESat-2 for characterizing vegetation structural damages at regional scales. Our specific objectives were to:

1. Evaluate the performance of machine learning (ML) models, specifically Random Forest (RF) and Extreme Gradient Boosting (XGBoost), for estimating canopy height across the hurricane-impacted landscapes using ICESat-2 data in combination with Sentinel-2 imagery, derived vegetation indices, and ancillary geospatial datasets.
2. Generate spatially contiguous, 30-m resolution canopy height maps for both pre-hurricane (2020) and post-hurricane (2021) periods using the better-performing canopy height prediction model.
3. Quantify canopy height changes across forested areas using pre- and post-hurricane maps and delineate damaged areas using combined structural (i.e., ICESat-2-derived maps) and thematic products (land cover and tree canopy).

## Materials and methods

**Fig. 1:**
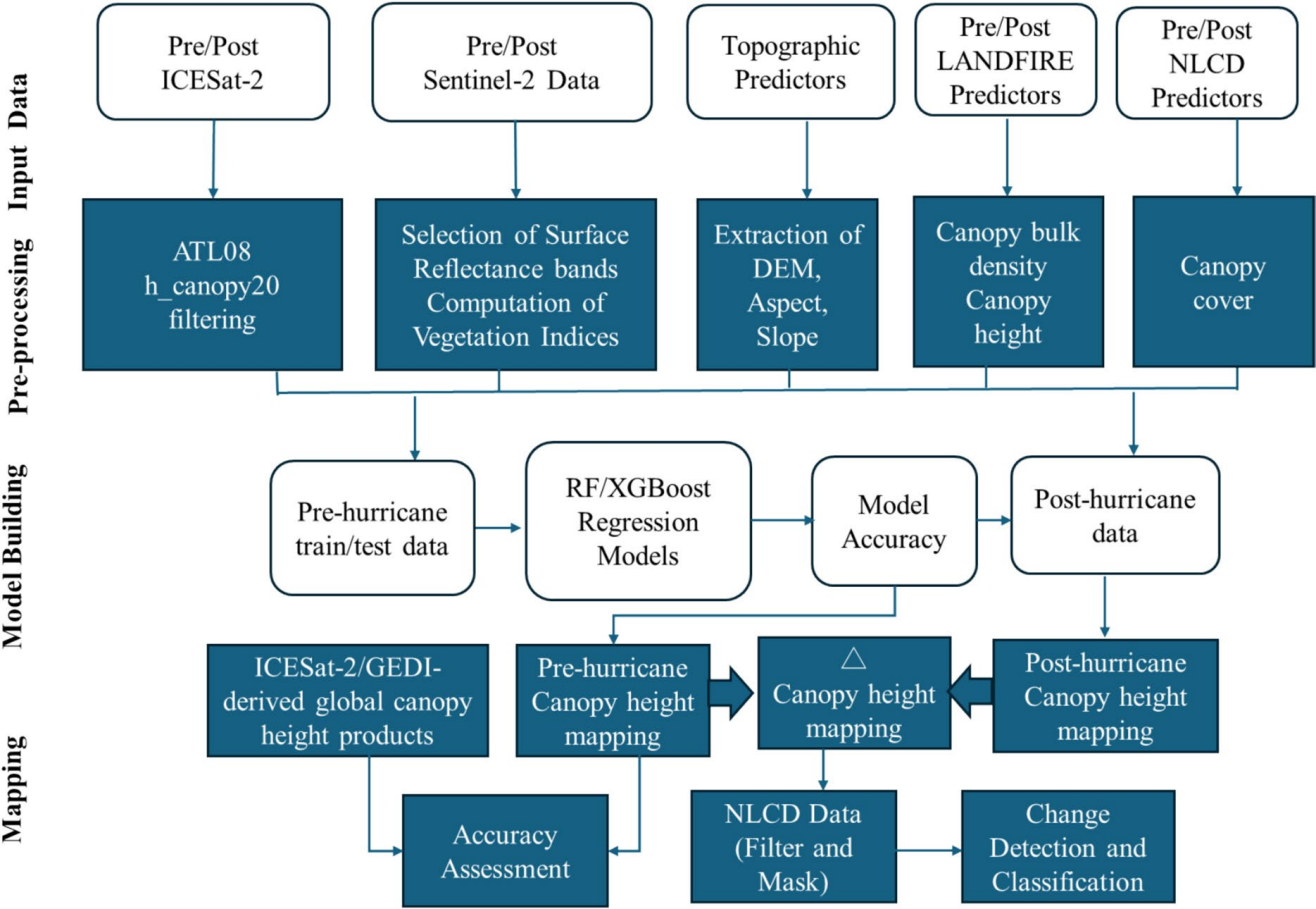
Flowchart showing research methodology

The methodological framework for this study consisted of four key stages, as illustrated in Fig. 1. First, multi-source input data were acquired, including 98th percentile canopy height (RH98) from ICESat-2 ATL08 sub-segments, Sentinel-2 surface reflectance bands and derived vegetation indices, LANDFIRE’s canopy height (CH) and canopy bulk density (CBD), and topographic variables (elevation, slope, aspect). Pre-hurricane data were acquired from January to August 2020, and post-hurricane data from January to August 2021. Second, predictor datasets were preprocessed and resampled to a common 30-m resolution to ensure spatial consistency and coverage. In the third stage, machine learning models were trained to predict canopy height using the pre-hurricane input data. Model accuracy was evaluated using standard performance metrics, including the coefficient of determination (R^2^), root mean square error (RMSE), and mean absolute error (MAE), based on a hold-out validation approach. In the final stage, the better-performing model was applied to both pre- and post-hurricane predictors to generate spatially continuous canopy height maps, and the differences between estimates were used to estimate canopy height changes across the study area. The pre-hurricane canopy height map was compared with existing ICESat-2 (National Snow and Ice Data Center) and GEDI (Global Forest Canopy Height, 2019 | GLAD) canopy height products. The NLCD land cover and canopy cover dataset were then used to extract forest-specific changes and assess structural damage and canopy cover changes resulting from impact of Hurricane Sally.

### Study area

**Fig. 2:**
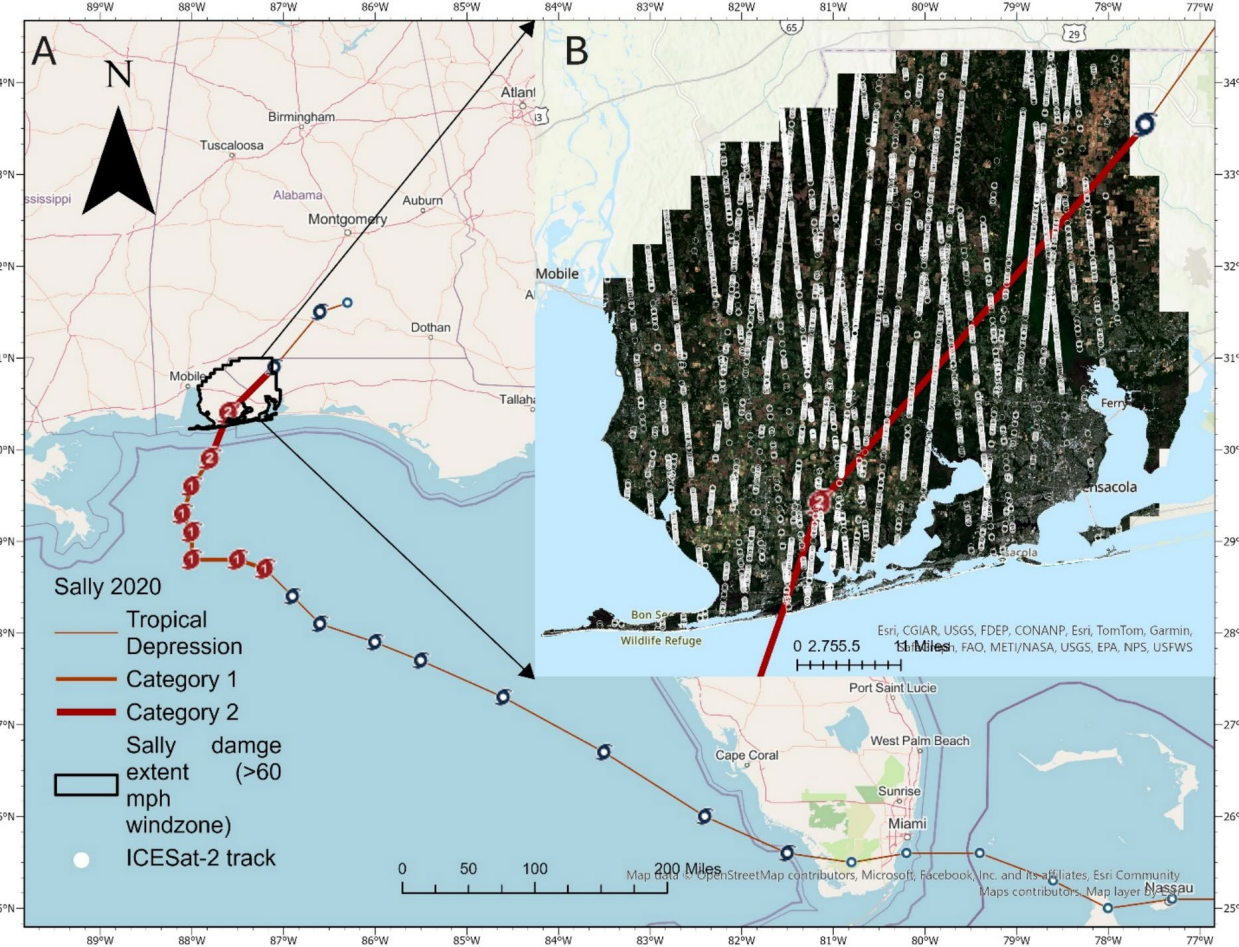
Study area map showing (**A**) Hurricane Sally’s track of landfall, intensity, maximum damaged zone with sustained windspeed greater than 60 mph, and (**B**) ICESat-2 tracks and track of hurricane landfall across the study extent overlaid on a Sentinel-2 true-color composite

The southeastern US, particularly the coastal regions of Alabama and Florida, are frequently impacted by hurricanes that cause widespread and recurring structural forest losses (Cole et al., 2021; NOAA, 2023; Sharma et al., 2021). For this study, we focused on the coastal counties of Alabama and Florida, specifically within Hurricane Sally’s maximum wind impact zone (> 65 mph) defined by the National Oceanic and Atmospheric Administration (NOAA). Hurricane Sally, a Category 2 storm with sustained winds reaching 110 mph, made landfall along the Alabama coast and the Florida Panhandle in September 2020, resulting in extensive damage estimated at $7.93 billion (NOAA, 2021a, 2021b). The Alabama Forestry Commission (AFC) reported that in Baldwin County alone, approximately 2440 acres of forest were affected, with 79,175 tons of timber lost, valued at around $1.5 million USD (AFC, 2020). These estimates were primarily derived from rapid post-event field assessments and localized inventory data. While valuable, such assessments underestimate the broader extent and variability of structural forest damage across the full extent of the hurricane-impacted region. This study extent (Fig. 2) represents the core disturbance zone where significant canopy damage occurred due to hurricane’s immediate high winds, structural damage, and long-term sustained damages from waterlogging, salinity stress, loss of regeneration leading to tree mortality (NOAA, 2021a). This region is situated along the Gulf Coastal Plain, which is characterized by low elevation (0–100 m above sea level), humid subtropical climate, and annual precipitation ranging from 1300 to 1700 mm (SCIPP, 2022). These environmental conditions support a complex and heterogeneous forested landscape, shaped by variations in topography, hydrology, and coastal proximity (Goebel et al., 2001). The dominant forest types include evergreen forests (57.5%), primarily southern pines (longleaf pine (*Pinus palustris*), slash pine (*Pinus elliottii*), shortleaf pine (*Pinus echinata*), and loblolly pine (*Pinus taeda*)), woody wetlands (40.75%) dominated by swamp tupelo (*Nyssa biflora*) and water tupelo (*Nyssa aquatica*), and interspersed smaller patches of deciduous and mixed forests (Chen & Fraser, 2009; NLCD, 2019a).

### ICESat-2’s ATL08 data

ICESat-2 carries the Advanced Topographic Laser Altimeter System (ATLAS), a photon-counting lidar instrument operating at a wavelength of 532 nm (green) with a pulse repetition rate of 10 kHz, enabling near-continuous sampling of Earth’s surface at approximately 0.7-m intervals along-track (Neuenschwander et al., 2023; Neumann et al., 2019). ATLAS splits its single laser beam into six beams (three beam pairs) using a diffractive optical element. Each pair consists of a strong and weak beam (energy ratio 4:1), spaced 90 m apart across-track and ~ 3 km between beam pairs (Markus et al., 2017). Data are collected along ground tracks (GTs) that correspond to these beams in the along-track direction. Canopy height estimates were derived from ICESat-2 ATL08 Version 006 product, which provides vegetation parameters at fixed 100 m and 20 m step-sizes along ICESat-2 tracks.

The ATL08 version 6 provides canopy height estimates derived from photon returns aggregated over 100 m × 14 m segments and 20 m × 14 m sub-segments within vegetated surfaces (Neuenschwander et al., 2022). In this study, we utilized the 98th percentile canopy height metric (h_canopy20 or RH98) from ATL08 sub-segments as a representation of top of canopy height. We selected data from strong beams only based on studies demonstrating the utility of this parameter for forest applications (Chen et al., 2023; Li et al., 2020; Narine et al., 2020; Neuenschwander et al., 2020; Rai et al., 2024). To minimize spatial autocorrelation, we extracted only the central 20-m value from each sub-segment, following the approach by Malambo and Popescu (2024) for mapping canopy height with ICESat-2. We filtered canopy height values to exclude extreme height values and non-vegetated observations, retaining canopy height values less than 45 m within the NLCD forest classes (Malambo & Popescu, 2024; Malambo et al., 2022). A total of 7484 subsegment canopy height data points were retained for model building within the NLCD forest classes after pre-processing and filtering 83,447 subsegments. We downloaded ATL08 data from the National Snow and Ice Data Center (NSIDC) website and collected across multiple satellite cycles from January to September 2020 for pre-hurricane analysis, and from January to September 2021 for post-hurricane analysis.

### Sentinel-2 imagery and imagery-derived metrics

Sentinel-2 is a multispectral imaging mission developed by the European Space Agency (ESA), comprising twin satellites: Sentinel-2A and Sentinel-2B, with a wide swath and high spatial resolution (10 m for RGB bands and NIR band, 20 m for red edge bands and Shortwave Infrared (SWIR) bands, 60 m for QA60 atmospheric correction bands) (Drusch et al., 2012). The mission supports land surface monitoring, particularly for vegetation, water, and soil conditions, across multiple spatial and temporal scales. A key advantage of Sentinel-2 over other freely available multispectral sensors, such as Landsat-8, is the inclusion of red-edge bands, which have been shown to be especially useful in vegetation disturbance detection and damage assessment (Immitzer et al., 2016). In this study, we used Level-2A surface reflectance data from the Harmonized Sentinel-2 MSI: Multispectral Instrument (SR) collection available in Google Earth Engine (GEE) (Gorelick et al., 2017). We selected scenes with less than 5% cloud cover for both the pre-hurricane (April–August 2020) and post-hurricane (April–August 2021) periods, corresponding to the leaf-on season.

We extracted a total of nine image bands for analysis: B02 (Blue), B03 (Green), B04 (Red), B05 (Red Edge 1), B06 (Red Edge 2), B07 (Red Edge 3), B08 (NIR), B11 (SWIR 1), and B12 (SWIR 2). The red edge bands (B05 and B06) and the SWIR bands (B11 and B12) were used as predictors based on studies showing improved model accuracy in structural vegetation monitoring (Xi et al., 2022). We used other image bands (RGB and NIR) independently to compute several vegetation indices (VIs) as shown in Table 1. Among these, the Normalized Difference Vegetation Index (NDVI), Enhanced Vegetation Indices (EVI) and Soil Adjusted Vegetation Index (SAVI) were selected for their proven relevance in modeling vegetation productivity (Rondeaux et al., 1996), aboveground biomass (Narine et al., 2020), and disturbance severity related to canopy stress (Aljahdali et al., 2021; Konda et al., 2018). We filtered cloud-affected pixels using the QA60 bitmask band and resampled all spectral bands and derived metrics to a 30-m grid size. We implemented all pre-processing, cloud masking, resampling, and VI calculations in GEE, enabling efficient extraction of high-quality, temporally consistent datasets for model input.

**Table 1:** Table showing VIs selected for predicting canopy height with ICESat-2

Vegetation Indices (VIs)	Description	Formula	Reference
Normalized Difference Vegetation Index (NDVI)	Assesses vegetation greenness and is used in monitoring plant health	(NIR − R)/(NIR + R)	(Singh et al., 2022)
Green Normalized Difference Vegetation Index (GNDVI)	Uses the green band to enhance sensitivity to chlorophyll content and vegetation stress detection	(NIR − G)/(NIR + G)	(Khunrattanasiri, 2022)
Soil-Adjusted Vegetation Index (SAVI)	Adjusts NDVI for soil brightness, improving sensitivity in areas in sparse vegetation like coastal forests	1.5*((NIR-R)/(NIR + R + 0.5))	(Khunrattanasiri, 2022)
Enhanced Vegetation Index (EVI)	Improves sensitivity to canopy structure and reduces soil and atmospheric influences	2.5 × ((NIR – R)/(NIR + 6R – 7.5B + 1))	(Gnilke & Sanders, 2022)
Green Chlorophyll Index (GCI)	Estimates canopy chlorophyll useful in assessing vegetation stress and productivity	(NIR/G) – 1	(Raman et al., 2025)
Modified Soil-Adjusted Vegetation Index (MSAVI)	Enhances SAVI’s effectiveness by removing background influences	(2 × NIR + 1 − sqrt((2 × NIR + 1)2 − 8 × (NIR − R)))/2	(Rondeaux et al., 1996)
Optimized Soil-Adjusted Vegetation Index (OSAVI)	Optimized by using fixed correction factor	(NIR—Red)/(NIR + Red + 0.16)	(Rondeaux et al., 1996)

### Ancillary datasets

In this study, we integrated several ancillary geospatial datasets to support canopy height modeling and disturbance assessment. These included vegetation structure products from the LANDFIRE, canopy cover data from the NLCD, and topographic variables derived from the Shuttle Radar Topography Mission (SRTM) as described in Table 2.

**Table 2:** List of ancillary predictors used in the study

Datasets	Description	Reference
2019/2022 LANDFIRE CH	Average height of top of the canopy derived from Landsat with ancillary variables at a 30-m grid size	(LANDFIRE, 2024)
2019/2022 LANDFIRE CBD	Density of available dead/non-photosynthetic biomass, estimated for 30-m pixels across forest classes only	(LANDFIRE, 2024)
2019/2021 NLCD canopy cover	Percentage tree canopy estimates across forest landcover classes at 30 m	(NLCD, 2019b)
SRTM DEM	30-m global terrain elevation dataset	(Farr et al., 2007)
Slope	Steepness of land surface calculated from DEM	Derived from SRTM; (Farr et al., 2007)
Aspect	Direction of slope faces derived from DEM	Derived from SRTM; (Farr et al., 2007)

The LANDFIRE program, managed by the U.S. Department of Agriculture and Forest Service, provides nationally consistent, 30-m resolution raster datasets to support wildfire management and ecological assessments (LANDFIRE, 2024). From this program, we extracted two key vegetation structure variables, Canopy Height (CH) and Canopy Bulk Density (CBD), for both pre-hurricane (2019) and post-hurricane (2022) conditions. These predictors have been shown to significantly improve large-area canopy height modeling (Malambo & Popescu, 2024). We downloaded all data from the official LANDFIRE portal and processed at their standard 30-m spatial resolution.

The National Land Cover Database (NLCD), developed by the U.S. Geological Survey and the Multi-Resolution Land Characteristics (MRLC) consortium, provides land cover products at a 30-m resolution using the modified Anderson Level II classification system consisting of 16 land cover classes (Wickham et al., 2021). We used the land cover dataset as a mask to extract canopy height values within the forest classes only. Additionally, the MRLC also provides tree canopy cover products since 2011. We used the 2019 and 2021 canopy cover layers as continuous predictors for pre- and post-hurricane canopy height modeling, respectively. These products are generated using multispectral satellite imagery like Sentinel-2 and Landsat, ground-based measurements using photo-interpretation of high-resolution aerial imagery, and other ancillary datasets (Toney et al., 2010).

We derived topographic variables from the SRTM v3 Digital Elevation Model (DEM), which offers global elevation data at 1 arc-second (~ 30 m) resolution (Farr et al., 2007). We assessed the DEM through Google Earth Engine (GEE) and clipped it to the study area. Using the DEM, we calculated slope and aspect, as these variables are known to influence vegetation distribution, growth, and structural variation across landscapes (Wang et al., 2018).

### Canopy height modeling

To model spatial canopy height variation, we employed two machine learning algorithms: RF and XGBoost to relate ICESat-2-derived canopy height estimates to multi-source remote sensing predictors. We trained both models in Python (*RandomForestRegressor* from the *scikit-learn* library and *XGBRegressor* from the *xgboost* library) using ICESat-2 RH98 values as the dependent variable, with Sentinel-2, LANDFIRE and ancillary predictors as the independent variables.

The RF model is an ensemble-based algorithm composed of multiple decision trees trained using bootstrap aggregation (bagging), where each tree learns from a random subset of the training data (Belgiu & Drăguţ, 2016). Predictions for non-training samples are obtained by averaging the outputs from all trees. RF is widely used in remote sensing due to its robustness against overfitting and ability to handle high-dimensional datasets. We optimized the RF model using hyperparameter tuning through a randomized search (Bergstra et al., 2012). Optimization involved randomly sampling parameter combinations from a defined hyperparameter grid: number of trees (*n_estimators*: 100, 300, 500), maximum tree depth (*max_depth*: 10, 20, 30, 40), minimum samples for splitting nodes (*min_samples_split*: 2, 5, 10), minimum samples per leaf (*min_samples_leaf*: 1, 2, 4), maximum features per split (*max_features*: 'sqrt', 'log2', None), and bootstrap sampling (*bootstrap*: True, False). We used fivefold cross-validation to identify the combination of parameters minimizing prediction error and enhancing model generalization.

We selected XGBoost, which is a regularized form of gradient-boosted decision trees, given its demonstrated superior performance in canopy height and vegetation structure modeling (Malambo & Popescu, 2021, 2024). Unlike RF, XGBoost builds trees sequentially, optimizing residual errors at each step and using second-order gradients to improve learning. The XGBoost regression model was configured using targeted hyperparameters adapted from Malambo and Popescu (2024). Model training incorporated tenfold cross-validation to enhance model stability and predictive accuracy. Key hyperparameters included a low *learning rate* (0.01) and a large *number of trees* (983), facilitating gradual, detailed learning from the data. Additionally, we implemented a deep tree structure (*max_depth* = 24) to effectively model complex interactions between canopy height and predictor variables. To reduce overfitting and improve generalization, we introduced controlled randomness through multiple subsampling parameters (*subsample*, *colsample_bytree*, *colsample_bylevel*, *colsample_bynode*).

For both RF and XGBoost models, we analyzed feature importance using SHAP (Shapley Additive exPlanations) values (Lundberg & Lee, 2017), which quantify the contribution of each predictor to the model’s output. This enabled both global and local interpretability of variable influence on canopy height prediction. We randomly split the dataset into 70% for training and 30% for testing to validate model performance. We evaluated model performance by using cross validation metrics to assess the stability and generalizability of both models. The coefficient of determination (R^2^), root mean square error (RMSE), and mean absolute error (MAE), were computed as defined in Eqs. (1)–(3), where *p*_*i*_ is the i^th^ predicted canopy height and *o*_*i*_ is the i^th^ observed canopy height and *n* is the number of samples.

1 $${\text{R}}^{2}=1-\frac{{\sum }_{\text{i}=1}^{\text{n}}{\left({\text{o}}_{\text{i}}-{\text{p}}_{\text{i}}\right)}^{2}}{{\sum }_{\text{i}=1}^{\text{n}}{\left({\text{o}}_{\text{i}}-\overline{\text{o} }\right)}^{2}}$$

where ($\overline{\text{o} }$) is the mean of the observed values.

2 $$\text{RMSE }= \sqrt{\left(\frac{1}{\text{n}}\right) {{\sum }_{\text{i}=1}^{\text{n}}\left({\text{p}}_{\text{i}}-{\text{o}}_{\text{i}}\right)}^{2}}$$

3 $$\text{MAE }= \left(\frac{1}{\text{n}}\right){\sum }_{\text{i}=1}^{\text{n}}\left|{\text{p}}_{\text{i}}-{\text{o}}_{\text{i}}\right|$$

### Pre- and post-hurricane canopy height mapping

To generate spatially continuous canopy height estimates across the study area, we applied the best-performing machine learning model trained on pre-hurricane ICESat-2 canopy height data with multi-source predictors using a Python-based geospatial workflow. We processed all spatial predictor layers, including Sentinel-2 bands, vegetation indices, LANDFIRE canopy metrics, and topographic attributes to a consistent 30-m spatial resolution, stacked into a multi-band input array. Using this model, we first generated wall-to-wall canopy height predictions for the pre-hurricane period (2020) and then applied the trained model to produce corresponding canopy height maps using post-hurricane ICESat-2 canopy height and corresponding predictors (2021). To focus the analysis on forested areas only, we masked both the pre- and post-hurricane canopy height rasters using NLCD forest class, ensuring that results reflected changes within forested land cover types only. This masking step was essential for isolating hurricane-induced structural change and excluding non-forested pixels from further analysis.

### Comparison of predicted canopy heights with existing products

To evaluate the accuracy of the predicted pre-hurricane canopy height map, we used two published canopy height products: (1) a global canopy height product derived from the GEDI (Global Ecosystem Dynamics Investigation) for the year 2019 by Potapov et al. (2021), and (2) an ICESat-2 derived canopy height map for contiguous US for the year 2020 by Malambo and Popescu (2024). GEDI is a full-waveform spaceborne lidar instrument onboard the International Space Station (ISS), collecting vegetation structure data between approximately 51.6° N and 51.6° S from April 2019 (Dubayah et al., 2020). However, data collection has not been continuous; there have been multiple storage periods during which the instrument was powered off due to ISS operational constraints (GEDI Science Team, 2024; GEDI Timeline). By integrating GEDI waveforms with optical metrics derived from Landsat Analysis Ready Data, a 30-m global forest canopy height map for 2019 was produced (Potapov et al., 2021). We accessed this dataset via the Global Land Analysis and Discovery (GLAD) platform (https://glad.umd.edu/dataset/gedi). Malambo and Popescu (2024) developed canopy height for the contiguous US using ICESat-2 and gap-filled Landsat and LANDFIRE predictors for the year 2020; we accessed this data through the project’s Google Drive (v1—Google Drive).

Following the approach of Xi et al. (2022), we performed cross-validation between the RF-modeled pre-hurricane canopy height with GEDI-derived global canopy height and ICESat-2-derived contiguous US canopy height within the forested land cover classes. To ensure both datasets were spatially aligned, we resampled to a consistent 30-m resolution and reprojected to a common coordinate reference system. The validation was carried out in Python environment (*rasterio library),* where we made pixel-level comparisons to assess the agreement between two rasters. Validation metrics include Pearson’s correlation (R), mean absolute error (MAE), root mean square error (RMSE).

### Canopy height change detection and thematic change assessment

To focus the analysis on forested ecosystems, we used the NLCD land cover dataset to mask out non-forested areas. We retained only pixels classified as Deciduous Forest (41), Evergreen Forest (42), Mixed Forest (43), and Woody Wetlands (90) for change analysis. We calculated the canopy height difference for each pixel using Eq. (4).

4 $$(\Delta \text{height})\text{i}\hspace{0.17em}=\hspace{0.17em}(\text{post}\_\text{height})\text{i}\hspace{0.17em}-\hspace{0.17em}(\text{pre}\_\text{hieght})\text{ where},\text{ i},\text{ i}\hspace{0.17em}=\hspace{0.17em}1, 2,...,$$

Negative values of Δheight indicate canopy height loss, interpreted as structural damage due to windthrow, stem breakage, or defoliation, while positive values represent gain, potentially due to vegetation regrowth or seasonal variability. To classify disturbance severity, we applied the following rule-based thresholds based on the literature (Ceccherini et al., 2023; Leitold et al., 2021; Mulverhill et al., 2022):

1. No change: −1m ≤|Δheight|< 1 m
2. Moderate loss: − 5 m ≤ Δheight <  − 1 m
3. Severe loss: Δheight <  − 5 m
4. Gain or regrowth: Δheight ≥ 1 m

We further analyzed the mean canopy height changes in NOAA-designated wind impact zones to assess the interplay between vegetation structural losses and storm severity. Additionally, we analyzed existing land cover classes and canopy covers pre- and post-hurricane (NLCD land cover and canopy cover 2019/2021) to describe the thematic changes in forest structure and cover driven by hurricane.

## Results

### Performance evaluations of canopy height models

**Fig. 3:**
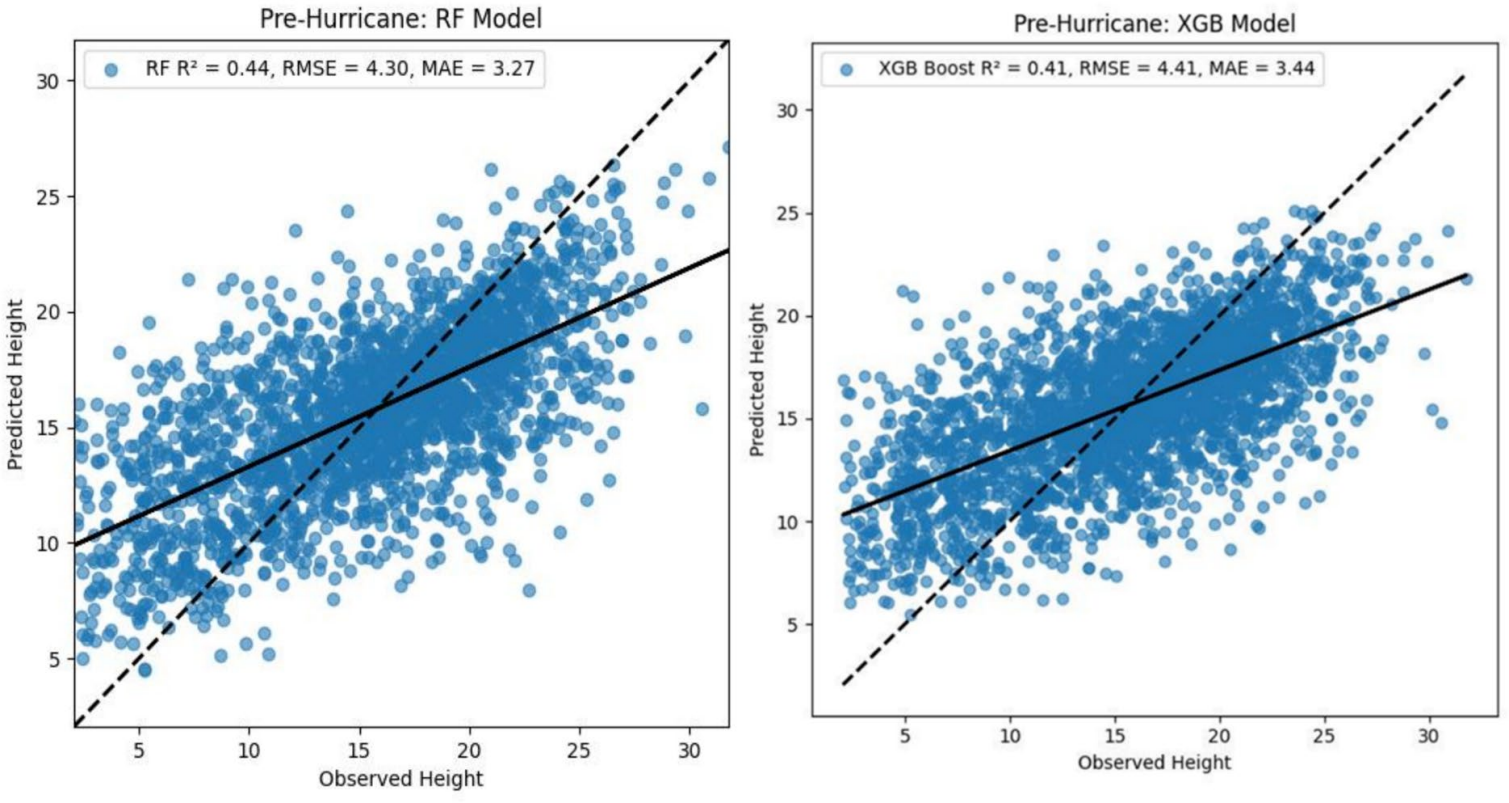
Scatterplots showing model-predicted canopy height vs ATL08 canopy height and accuracy of (**a**) RF model, and (**b**) XGBoost regression model with pre-hurricane dataset

The optimized RF model achieved moderate prediction accuracy (R^2^ = 0.44, RMSE = 4.30 m, MAE = 3.27 m) on the withheld test data (Fig. 3a). When evaluated on withheld test data, the XGBoost model demonstrated moderate predictive performance (R^2^ = 0.41, RMSE = 4.41 m, MAE = 3.44 m) (Fig. 3b).

**Fig. 4:**
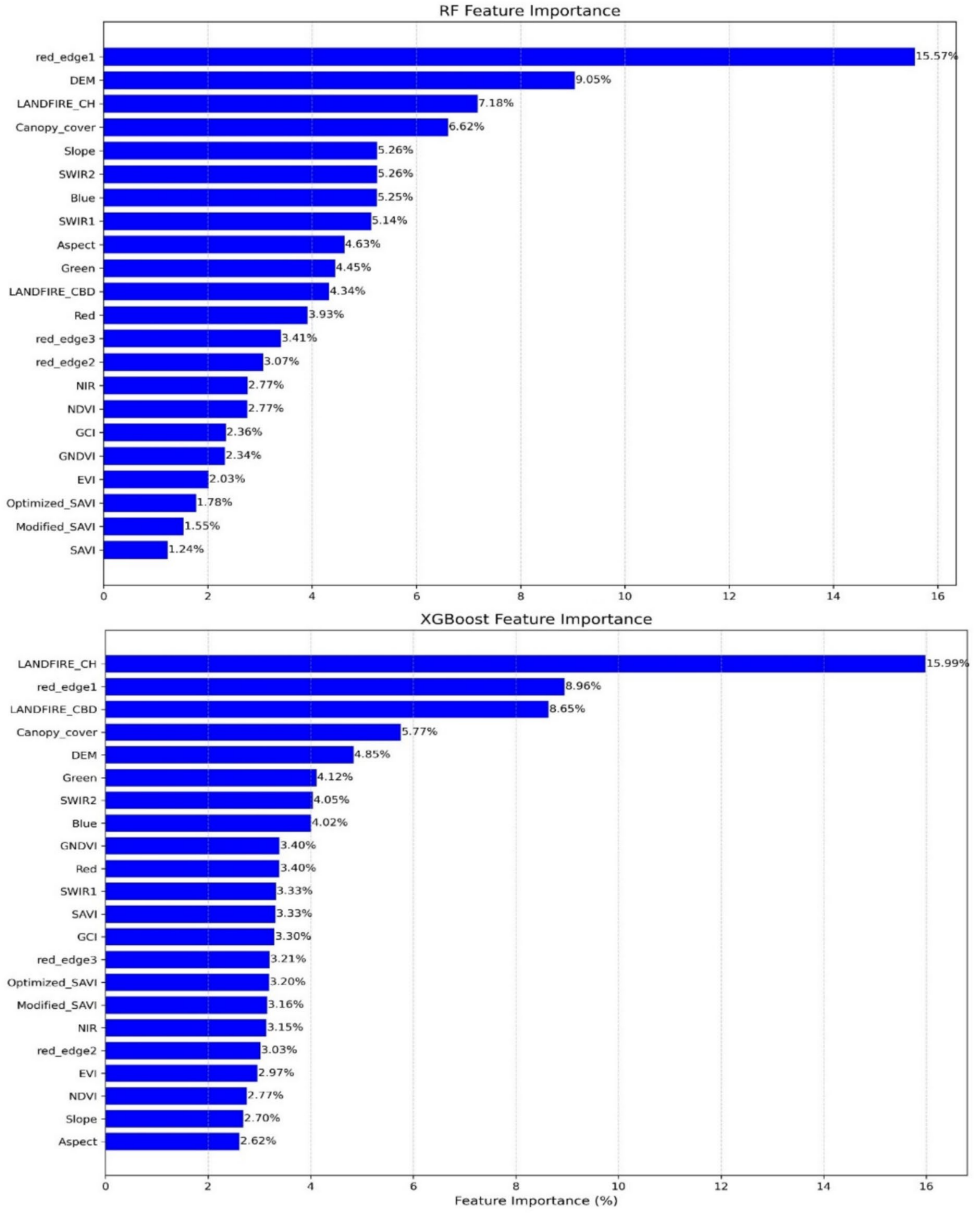
Plot showing variable contributions in the (**a**) RF model and (**b**) XGBoost regression model for pre-hurricane canopy height modelling

For the RF model, feature importance analysis identified Sentinel-2’s red-edge band 1 (15.57%), DEM (9.05%), and LANDFIRE canopy height (7.18%) as the most significant predictors in model building (Fig. 4a). Whrease for the XGB model, the most significant predictors were LANDFIRE canopy height (15.99%) (CH), Sentinel-2 red-edge1 band (8.96%), and LANDFIRE canopy bulk density (8.65%) (Fig. 4b).

### Evaluation of modeled canopy heights with independent canopy height products

**Fig. 5:**
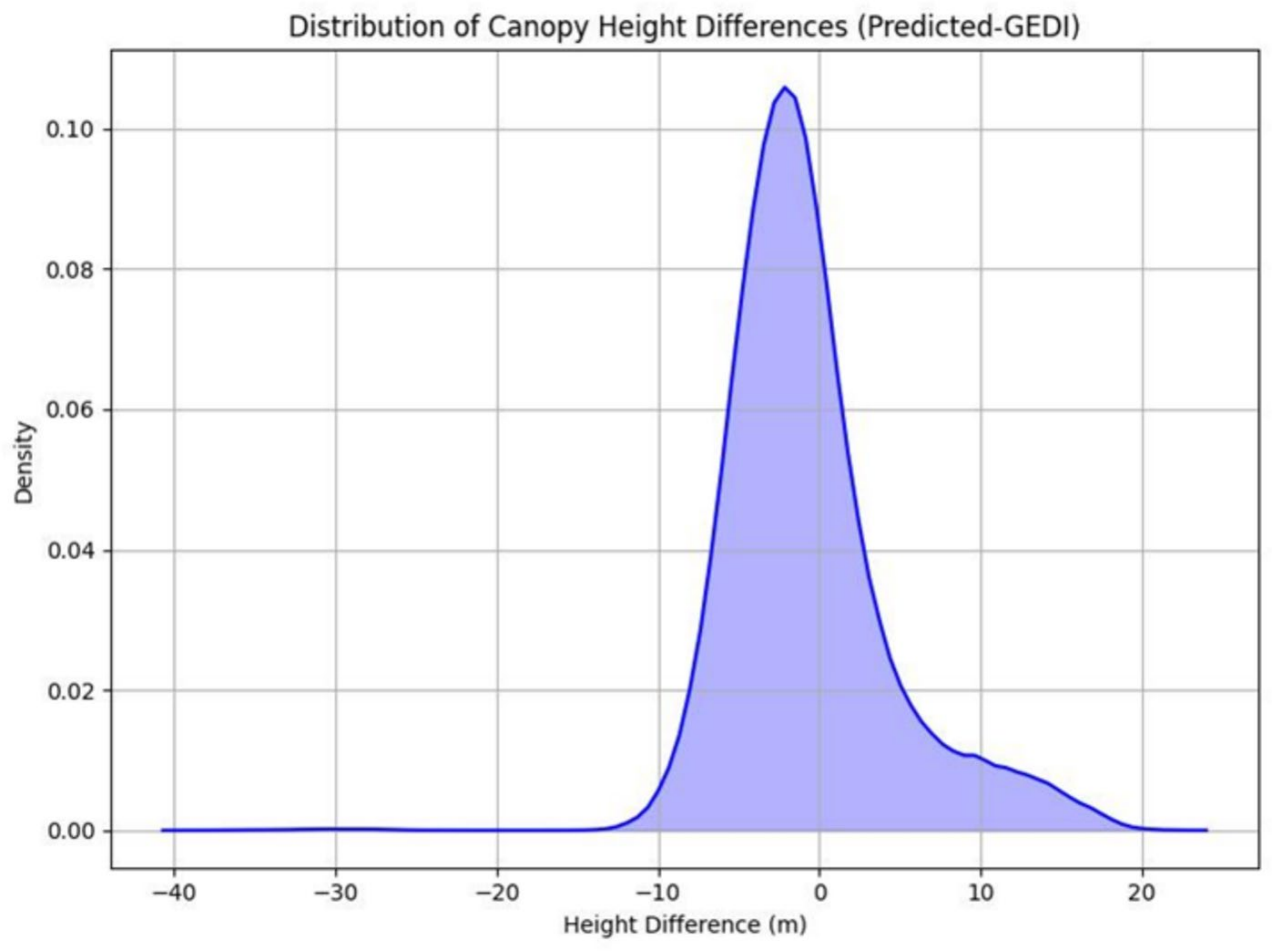
Density plot showing distribution of canopy height difference for predicted vs GEDI canopy height values

The accuracy of the pre-hurricane canopy height map generated by the best-performing model (RF) was evaluated by comparing it to independent, existing canopy height datasets: the GEDI-derived global canopy height map (2019) and the ICESat-2-derived contiguous US canopy height map (2020). This validation aimed to assess model performance and reliability in generating accurate, spatially explicit canopy height predictions across the study extent. The histogram of canopy height differences revealed a near-zero peak (Fig. 5), indicating minimal systematic bias between the predicted and reference canopy height products, thus confirming reasonable accuracy of the model-derived height map.

**Fig. 6:**
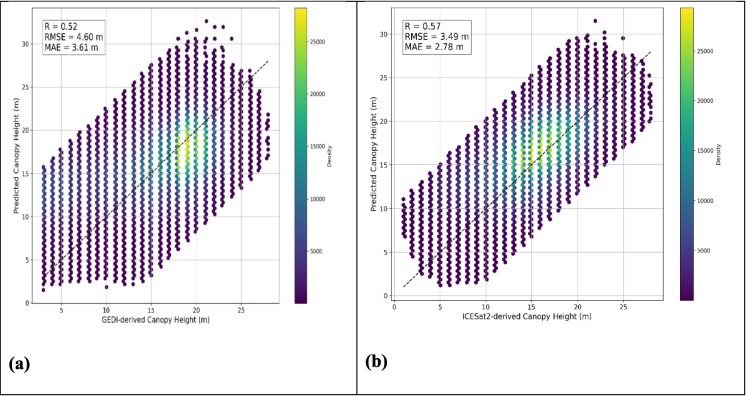
Comparison of model-predicted canopy height with existing canopy height products; (**a**) Model-predicted canopy height vs GEDI derived global canopy height 2019 by Potapov et al. (2020), and (**b**) Model predicted canopy height vs ICESat-2 derived global canopy height 2020 by Malambo and Popescu (2024)

Quantitative performance metrics showed a stronger agreement between the modeled canopy height and the ICESat-2-derived contiguous US map (R = 0.57, RMSE = 3.49 m, MAE = 2.78 m) compared to the GEDI-derived global canopy height product (R = 0.52, RMSE = 4.60 m, MAE = 3.61 m) (Fig. 6).

### Canopy height change mapping

**Fig. 7:**
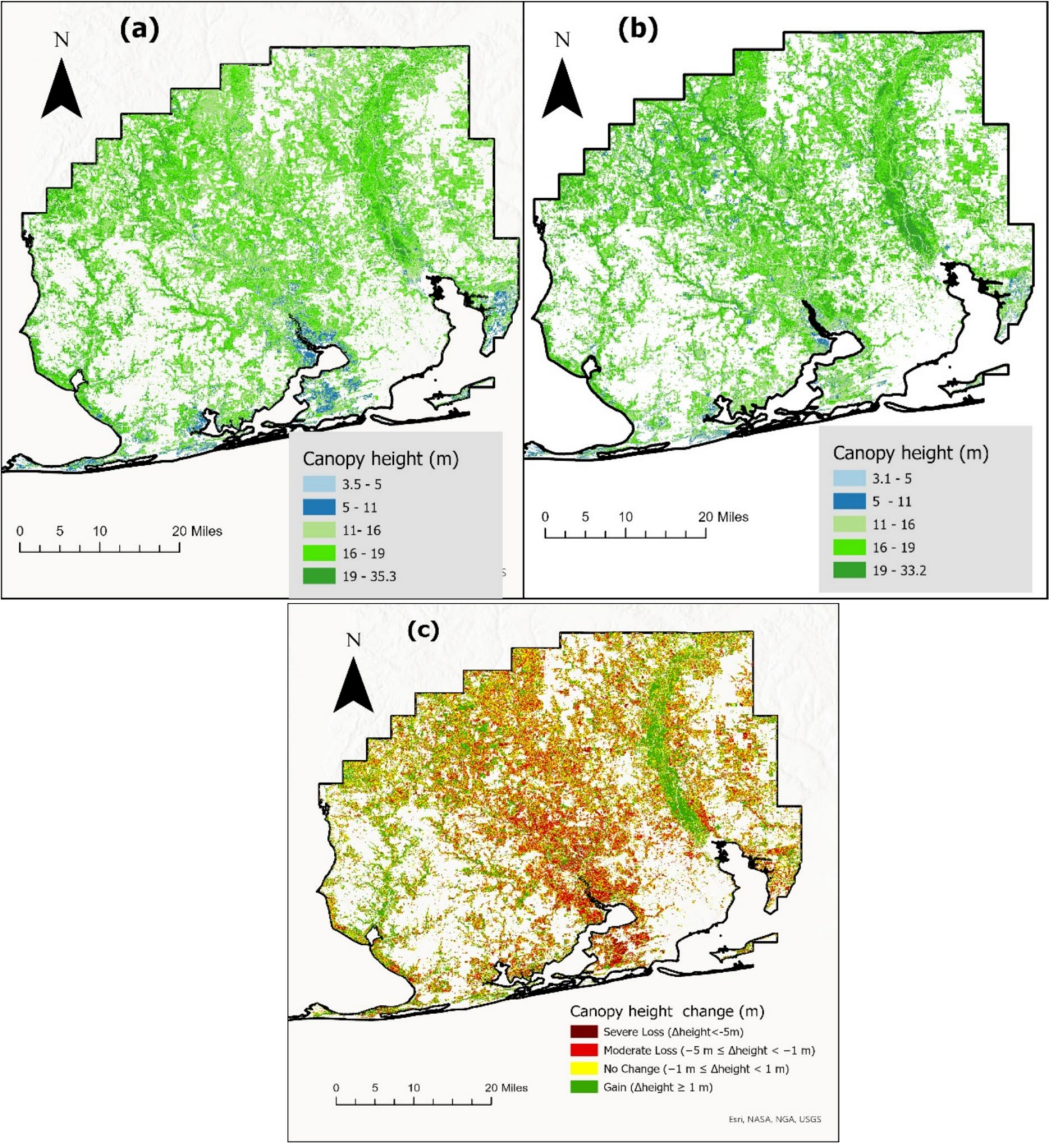
Map showing pre-hurricane forest canopy height map and post-hurricane canopy height map and canopy height differences within the extent of damage, (**a**) Predicted pre-hurricane canopy height map using RF model, (**b**) post-hurricane canopy height map using pre-hurricane trained model, (**c**) canopy height difference map post-hurricane

The canopy height difference map (ΔRH98) showed a clear spatial pattern of canopy height reduction associated with hurricane Sally, with the largest decreases, up to 15 m, observed near the landfall area along the coast of Alabama and Florida (Fig. 7). Canopy height changes were less pronounced farther inland, indicating reduced storm impact.

Land cover change analysis using NLCD, 2019a, 2019b and 2021 data revealed that areas of evergreen forest and woody wetlands experienced the most substantial structural transitions. Evergreen forests were predominantly converted into barren land, herbaceous cover, and scrub (Table 3), corresponding with average canopy height reductions of 5.2 m, 4.0 m, and 0.4 m respectively. Woody wetlands were primarily transformed into emergent herbaceous wetlands, with a mean canopy height loss of 2.3 m. Mixed forests were also converted into herbaceous land cover classes with a mean canopy height decrease of − 2.8 m (Table 3).

**Table 3:** Table showing land cover changes and associated canopy height changes post-hurricane

NLCD Forest Classes pre-hurricane (2019)	NLCD changed land cover classes post-hurricane (2021)	Mean Canopy Height Change (m)	Area Changed (ha)
Evergreen Forest	Barren Land	− 5.17	135
Evergreen Forest	Herbaceous	− 4.03	10,481.8
Evergreen Forest	Shrub/Scrub	− 0.43	672.6
Mixed Forest	Herbaceous	− 2.77	63.2
Woody Wetlands	Emergent Herbaceous Wetlands	− 2.30	1239.2

**Fig. 8:**
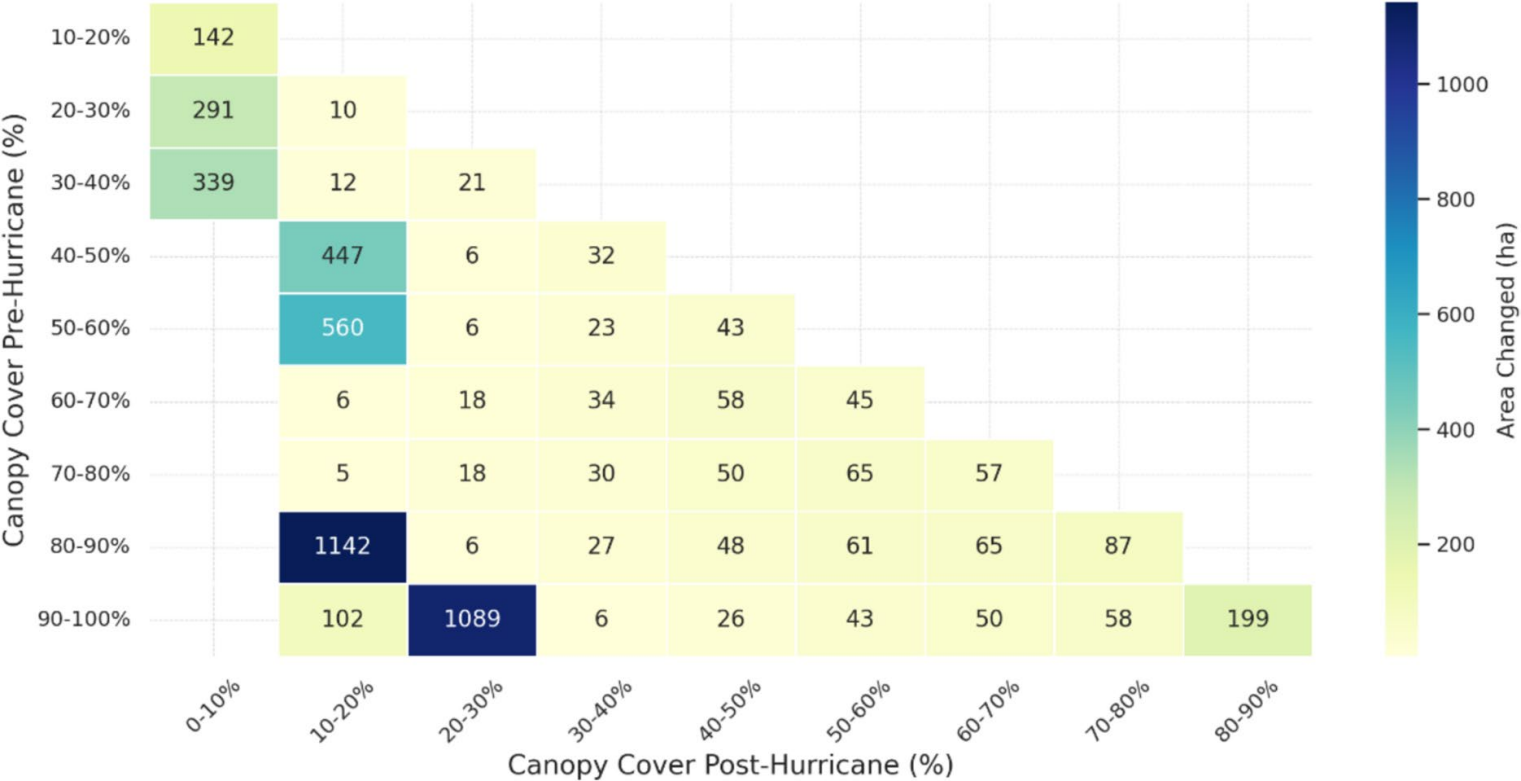
Plot showing associated area changes with canopy cover changes post-hurricane

**Fig. 9:**
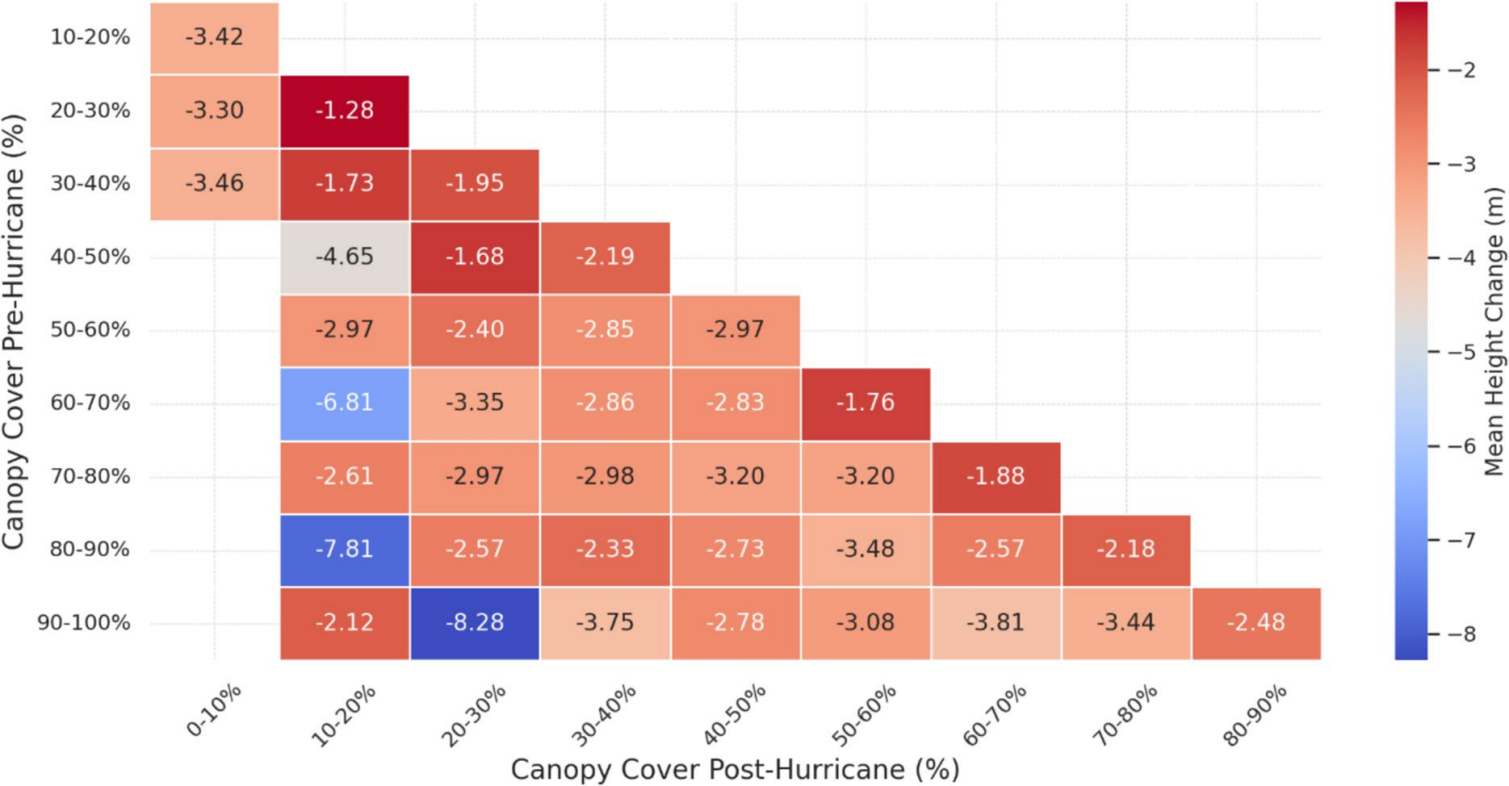
Plot showing associated mean canopy height changes with canopy cover changes post-hurricane

**Fig. 10:**
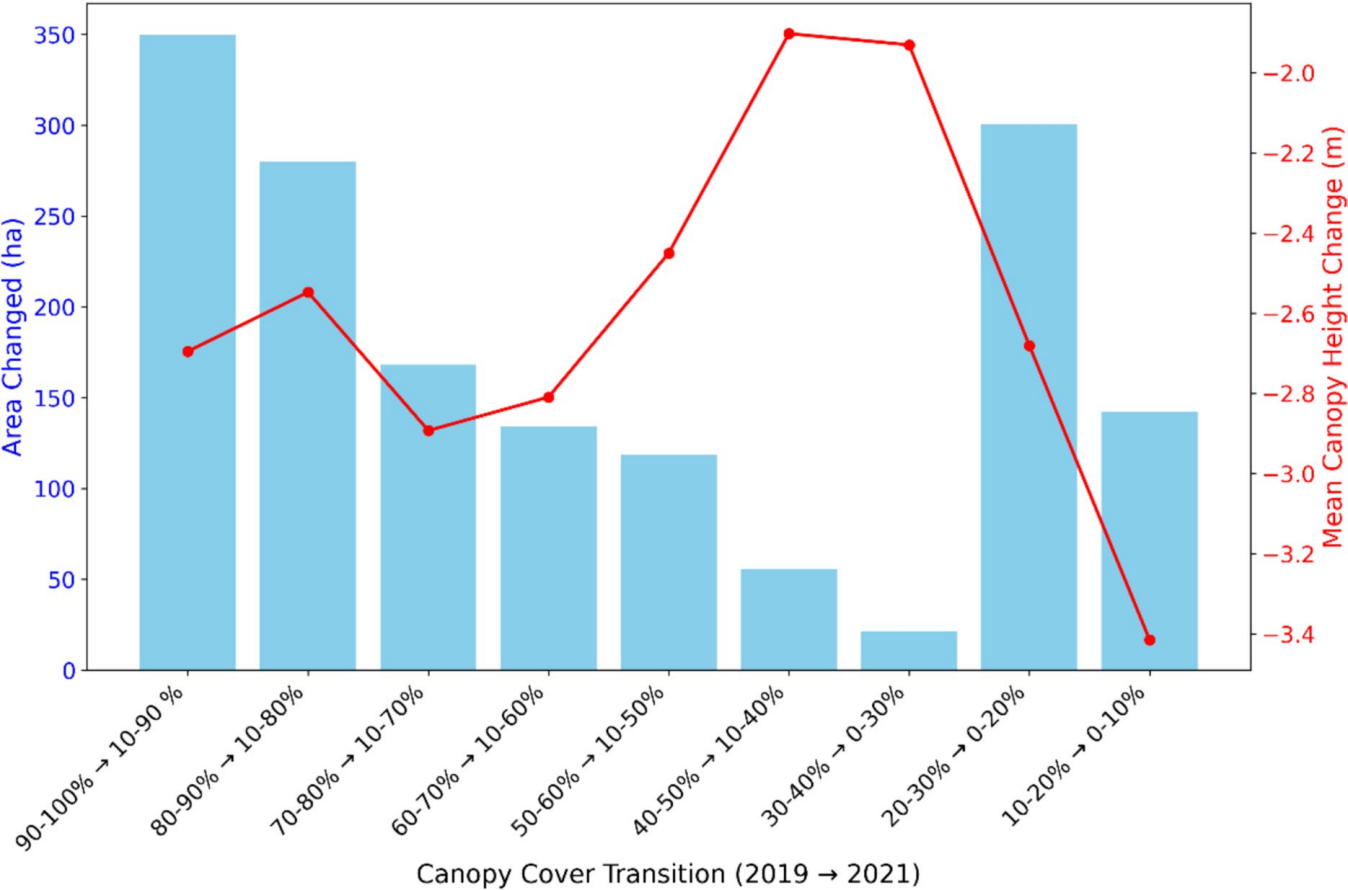
Plot showing changes in canopy cover after hurricane with pixel changes and height loss

Pre- and post-hurricane canopy cover comparisons using NLCD canopy cover 2019/2021 revealed that in terms of area changed, denser canopies (> 80%) were significantly reduced compared to moderate canopies, and the overall change in area was greater for denser canopies (Fig. 8). Complementing these findings, there was also a greater intensity of canopy height reduction (− 6 m to − 8.3 m) in areas where canopy cover substantially decreased to lower cover classes (Fig. 9). For instance, when pre-hurricane (80%–90%) canopy cover changed to post-hurricane (10%–20%) canopy cover classes, a mean canopy height decrease of 7.8 m was observed. Overall, the findings indicate that areas with high (> 60%) and low (< 30%) canopy cover experienced greater changes both in terms of area and canopy height loss, while areas with moderate canopy cover (30–60%) exhibited lower extent and canopy height losses (Fig. 10).

## Discussion

In this study, we evaluated a framework for determining hurricane-induced forest changes, using multi-temporal ICESat-2 observations in conjunction with Sentinel-2, LANDFIRE, NLCD, and SRTM-DEM data in coastal Alabama and Florida. This multi-source integration approach contributes to advancing the broader application of ICESat-2 in forest disturbance monitoring by extending its utility beyond conventional biomass estimation (Jiang et al., 2022; Narine et al., 2019, 2020) and terrain and canopy height mapping (Feng et al., 2023; Fernandez-Diaz et al., 2022; He et al., 2023; Liu et al., 2021a, 2021b; Malambo & Popescu, 2021; Neuenschwander et al., 2020; Neuenschwander & Magruder, 2019), and geophysical elevation change mapping (Csatho et al., 2019; Michaelides et al., 2021; Xu et al., 2023). By focusing on canopy height dynamics before and after a hurricane event (Hurricane Sally (2020)), the research examines the utility of combining ICESat-2 with vegetation and height-related predictors to quantify changes in canopy height changes across a heterogeneous, hurricane-affected coastal forest ecosystem.

The sparse availability of ICESat-2 coverage and variability of photon return quality in areas near the coast present challenges in modeling canopy height across such landscapes. Prior studies estimating canopy height using spaceborne lidar in similar coastal or disturbed environments have reported R^2^ values ranging from 0.30 to 0.55, RMSE values between 3.5m and 6.5 m (Dubayah et al., 2020; Lang et al., 2022; Malambo & Popescu, 2024). This study provides a similar range of R2 values ranging 0.41 (XGB) to 0.44 (RF) and a RMSE value ranging between 4.76 m (XGB) and 4.30 m (RF). These results are consistent with prior findings showing RF's effectiveness in capturing forest structural complexity, particularly in disturbed or heterogeneous environments (Xi et al., 2022; Zhang & Liu, 2023). While XGBoost has demonstrated better performance in estimating canopy heights at national scales like Malambo and Popescu (2024), comparatively lower accuracy is observed at this disturbance-focused, local-scale study. This underscores the model’s sensitivity to the spatial variability and sampling sparsity of ICESat-2 input data (Zhang & Liu, 2023). The lack of sufficient localized training data likely limited its ability to generalize effectively over coastal forests. For instance, over the eastern coastal extent of the study area (Fig. 2) (Escambia County, Florida), only 357 observations (357 20-m sub-segments) were available to model an area of 150 square miles.

In both machine learning models, it is worth noting that Sentinel-2’s red-edge band, elevation, and LANDFIRE variables emerged as top predictors. This finding aligns with prior studies that identify red-edge reflectance as a strong proxy for canopy structural attributes (Immitzer et al., 2016; Xi et al., 2022). LANDFIRE canopy height (CH) and canopy bulk density (CBD) were also major contributors for modeling canopy height, confirming the value of integrating canopy structural information to improve model performance in forest height prediction (Malambo & Popescu, 2024; Malambo et al., 2022). However, NIR band which is a core component in many vegetation indices used in both machine learning models appears lower ranked in the feature importance plot likely due to multicollinearity with VIs, making it difficult for the model to distinguish individual contribution to the output (Tian et al., 2023).

The better-performing RF-modeled pre-hurricane canopy height map was compared with two independent canopy height products: the GEDI-derived global canopy height map (2019) by Potapov et al. (2021) and the ICESat-2-derived contiguous US canopy height map (2020) by (Malambo & Popescu, 2024). The model showed stronger agreement with the ICESat-2-derived canopy height product (R = 0.57, RMSE = 3.49 m) compared to the GEDI-derived global canopy height product (R = 0.52, RMSE = 4.60 m). This is likely due to the temporal alignment; both predicted and reference canopy height maps were derived using data collected in 2020. Additionally, using a reference product based on the same ATL08 data reduces systematic discrepancies arising from geolocation differences, as previously noted by Markus et al. (2017) and Neumann et al. (2019). Favrichon et al. (2025) and Xi et al. (2022) further support that global-scale products are useful for validating local-scale modeling efforts, and temporally and sensor-consistent reference datasets provide stronger agreement.

The canopy height difference map (ΔRH98) revealed structural changes following Hurricane Sally, with canopy height reductions of up to 15 m concentrated at the region of hurricane landfall and around the realm of hurricane path. This spatial gradient is consistent with the hurricane impact path, where storm surge and high wind velocities exert the greatest pressure near landfall and within the travel path (Leitold et al., 2021). In contrast, interior forests showed relatively lower height loss and even localized signs of regrowth, a trend corroborated by studies in other post-hurricane landscapes (Zhu et al., 2024).

Further analysis using the NLCD land cover and canopy cover datasets pre- and post-hurricane, focusing on forested classes, revealed that evergreen forests and woody wetlands underwent the most substantial thematic transitions. The greatest area of land cover change was observed in evergreen forests transitioning to herbaceous classes, followed by shifts to scrub and barren land. Woody wetlands primarily transitioned into emergent herbaceous wetlands, indicating notable hydrological and vegetative shifts in wetland-dominated areas. These thematic transitions were accompanied by considerable structural changes in canopy height. Evergreen forests showed mean canopy height losses ranging from 2.8 m to 5.2 m, depending on the type of transition (e.g., to herbaceous, scrub, or barren classes), while woody wetlands experienced an average reduction of 2.3 m. These patterns are consistent with disturbance studies that highlight coniferous and wetland ecosystems as being particularly susceptible to structural collapse under stress (Leitold et al., 2021).

In terms of canopy cover, the most substantial changes were observed in areas with initially dense (> 60%) and thin (< 30%) cover, both of which underwent significant reductions in coverage. In contrast, moderately covered areas (30–60%) experienced relatively less change. This pattern was further reflected in the structural domain, where both dense and sparse canopy cover classes exhibited greater canopy height losses compared to areas with moderate canopy density. This dual pattern of canopy cover and structural decline aligns with previous findings by (Gough et al., 2022) who noted that both sparse and dense canopies are more likely to undergo structural realignment post-disturbance due to their exposure and canopy reorganization dynamics. Together, these results underscore the importance of integrating both canopy height and canopy cover into forest monitoring programs. While height captures vertical changes (e.g., windthrow, mortality), canopy cover highlights lateral changes (e.g., defoliation, crown loss), making them complementary indicators for post-disturbance structural assessments (Konduri et al., 2023; Queinnec et al., 2024).

Despite encouraging results, several limitations remain. First, the non-random sampling pattern and spatial sparsity of ICESat-2 tracks may result in underrepresentation of certain land types or forest conditions, as noted in Malambo and Popescu (2024)**,** resulting in observed moderate accuracy from the hold-out validation. The moderate accuracy achieved by the machine learning models likely reflects the complexity of modeling across a large, ecologically variable landscape with uneven sampling density. It is also important to acknowledge that an independent field-based validation dataset was not available due to the spatial sparsity of ICESat-2 footprints and time frame for pre-and post-dataset in hurricane impacted region. Consequently, the validation relied on the derived (GEDI 2019 global and ICESat-2 2020 US) canopy height products, which, while valuable, may not fully capture actual variability. Future studies could explore deep learning models trained on local ICESat-2 samples. Further work should also develop multi-source Earth Observation data fusion that integrates multi-spectral and radar data (e.g., Sentinel-1 backscattering coefficients and PALSAR observations) to mitigate cloud interference and enhance predictions in sparsely sampled regions.

## Conclusions

This study demonstrates the potential of integrating multi-temporal ICESat-2 observations with complementary remote sensing datasets to model and assess changes in coastal forests impacted by hurricanes. We used multi-temporal canopy height observations from ICESat-2, along with Sentinel-2 imagery-derived variables, LANDIRE’s canopy metrics, and elevation to detect and assess hurricane-driven structural, thematic, and cover changes. The novelty of this work lies in its use of temporally aligned canopy height and predictor datasets to develop spatially continuous, disturbance-specific multi-temporal canopy height maps. This approach broadens ICESat-2’s potential beyond biomass, canopy height, and elevation mapping. By jointly analyzing canopy height and canopy cover changes, the study reveals patterns of forest damage that are both structurally and thematically explicit. The impact of hurricanes was found to be stronger in regions dominated by evergreen forests, where woody wetlands were comparatively resistant. Similarly, greater impacts were examined for denser and sparse canopies, whereas moderate canopies were more resistant to structural damages. Such dual-dimension assessments provide a clearer understanding of disturbance severity across ecologically diverse landscapes, offering valuable insights for forest recovery, carbon dynamics, and long-term resilience planning in hurricane-prone regions. Importantly, this work reinforces the growing recognition of high-resolution, multi-temporal lidar and multispectral imagery and imagery-derived predictors as critical resources for post-disturbance forest assessment. The predictive performance achieved using machine learning models, particularly in hurricane-impacted regions, demonstrates the value of such methods for operational forest monitoring.

## Data Availability

The data used in the study are from entirely open-access sources. ATL08 data were downloaded from NASA Earth Data Search (Earthdata Search | Earthdata Search), Harmonized Seninel-2 Surface Reflectance (SR) product and derived Vegetation Indices (VIs) were retrieved from Google Earth Engine, Hurricane track and landfall data were downloaded from NOAA NHC Archive (NHC Data Archive), topographical variables were derived from SRTM-DEM (TNM Download v2), and LANDFIRE predictors were obtained from https://landfire.gov/data.
